# The complete mitochondrial genomes of *Laudakia Papenfussi* (Iguania; Agamidae)

**DOI:** 10.1080/23802359.2019.1644238

**Published:** 2019-07-22

**Authors:** Liang-Liang Dai, Li-Fang Peng, Yan-An Gong, Song Huang, Shun-Qing Lu

**Affiliations:** College of Life and Environment Sciences, Huangshan University, Huangshan, 245041, China

**Keywords:** Mitogenome, Papenfuss' rock agama, phylogeny

## Abstract

The Papenfuss' Rock Agama *Laudakia papenfussi* is an endemic species in the Tibet Autonomous Region of China. The complete mitochondrial genome sequence of this species was determined by shotgun sequencing. The total length of mitogenome is 17,005 bp and its base composition was 37.3% for A, 23.9% for T, 12.5% for G, and 26.3% for C, which contains 13 protein-coding genes, 22 tRNA genes, 2 ribosome RNA genes, and 2 control regions. The phylogenetic tree of *L*. *papenfussi* and 15 other related species was reconstructed using maximum likelihood (ML) methods. The mitogenome sequence presented here will be useful to understand the phylogenetic relationships of *Laudakia*.

The genus *Laudakia*7 contains 20 species with a checkered taxonomic history (Baig et al. 2012, Zou et al. 2016). China hosts six species of rock agamas, all of which are endemic to Tibetan Plateau and Xinjiang province: *L*. *sacra*, *L*. *wui*, *L*. *tuberculata*, *L*. *papenfussi*, *L*. *himalayana* and *L*. *stoliczkana*. *Laudakia papenfussi* was described by Zhao (1998) based on a sole specimen (adult male CIB 775001) was collected on 1 July 1976 from Zanda, Xizang (Tibet), China. No information on this species appeared until Zou et al. (2016) collected one female and six males at the type locality and described it. This species is a mountain-dwelling species (Baig et al. 2012). Although there are 20 species in the genus *Laudakia*, a few sequences of this genus have been determined. Three of these sequences are the complete mitochondrial genome of *L*. *tuberculata*, *L*. *sacra* , and *L*. *wui*. In this paper, we determined and described the mitogenome of *L*. *papenfussi* in order to obtain basic mitochondrial genetic information of this species.

The specimen of *L*. *papenfussi* was collected from Diya township (E78°45′22″, N31°49′0″; 2891 m), Zanda County, Ngari Prefecture, Tibetan Autonomous Region, China on 28 July 2017. It was preserved and deposited in the Museum of Huangshan University (Voucher numbers: HS17322). The total length of the complete mitogenome (Genbank accession number: MK585008) of *L*. *papenfussi* was sequenced to be 17,005 bp which consisted of 13 typical vertebrate protein-coding genes (PCGs), 22 transfer RNA (tRNA) genes, 2 ribosomal RNA (rRNA) genes, and 2 control regions (D-loop). The base composition was 37.3% for A, 23.9% for T, 12.5% for G and 26.3% for C. The positions of RNA genes were predicted by the MITOS (Bernt et al. 2013) and the locations of protein-coding genes were identified by comparing with the homologous genes of other related species. Most of the *L*. *papenfussi* mitochondrial genes are encoded on the H-strand except for the ND6 gene and eight tRNA genes, which are encoded on the L-strand. The gene order, contents, and base composition are identical to those found in typical vertebrates (Boore 1999; Sorenson et al. 1999; Zeng et al. 2019).

To further validate the newly determined sequences, whole mitochondrial genome sequences of *L*. *papenfussi* in this study and together with other 15 related species from GenBank was used to perform the phylogenetic analysis. We aligned these sequences using Clustal X (Thompson et al. 1997). Maximum likelihood (ML) methods were used to reconstruct phylogenetic tree in http://www.phylo.org/portal2/login. The phylogenetic analysis (Figure 1) result was consistent with the previous research. It is shown that the mitogenome of this species was genetically closest to that of *Laudakia tuberculata* with high support. It indicated that our new determined mitogenome sequences could meet the demands and explain some evolution issues.

**Figure 1.**
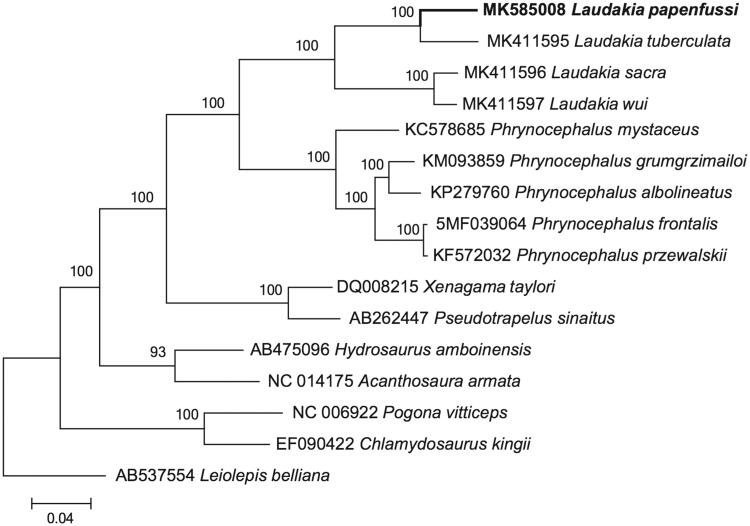
A maximum likelihood (ML) tree of *L. papenfussi* in this study and 15 other related species was constructed based on the dataset of the whole mitochondrial genome by online tool RAxML. The numbers above the branch meant bootstrap value. Bold black branches highlighted the study species and corresponding phylogenetic classification.
